# New Thoracotomy Closure, a Simple Way to Decrease Chronic Post-Operative Pain in Selected Patients—Blinded Prospective Study

**DOI:** 10.3390/jpm11101007

**Published:** 2021-10-06

**Authors:** Ioan Adrian Petrache, Cristian Oancea, Elisei Moise Hasan, Octavian Constantin Neagoe, Emanuela Tudorache, Mihaela Ionica, Ovidiu Nicolae Burlacu

**Affiliations:** 1 First Discipline of Surgical Semiology, First Department of Surgery, “Victor Babeș” University of Medicine and Pharmacy Timișoara, Eftimie Murgu Square No. 2, 300041 Timișoara, Romania; ioan.petrache@umft.ro; 2 Clinic of Thoracic Surgery, Emergency Clinical Municipal Hospital Timișoara, Gheorghe Dima Street No. 5, 300079 Timișoara, Romania; hasanelisei@yahoo.com; 3 Discipline of Pneumology, Department of Infectious Diseases, “Victor Babeș” University of Medicine and Pharmacy Timișoara, Eftimie Murgu Square No. 2, 300041 Timișoara, Romania; oancea@umft.ro (C.O.); emanuela.tudorache@umft.ro (E.T.); 4 Second Clinic of General Surgery and Surgical Oncology, Emergency Clinical Municipal Hospital Timișoara, Gheorghe Dima Street No. 5, 300079 Timișoara, Romania; dr.mihaela.ionica@gmail.com; 5 Second Discipline of Surgical Semiology, First Department of Surgery, “Victor Babeș” University of Medicine and Pharmacy Timișoara, Eftimie Murgu Square No. 2, 300041 Timișoara, Romania

**Keywords:** chronic pain, thoracotomy, chest closure, post-thoracotomy pain syndrome

## Abstract

Background and Objectives: Chronic post-thoracotomy pain syndrome (PTPS) is a very common and uncomfortable complication, occurring frequently after thoracic operations, leading to the necessity of further medication and hospitalizations. One important risk factor in developing chronic pain is the chest closure technique, which can lead to chronic intercostal nerve damage. This study proposes an alternative nerve-sparring closure technique to standard peri-costal sutures, aimed toward minimizing the risk of chronic pain in selected patients. Materials and Methods: We performed a prospective randomized study on 311 patients operated for various thoracic pathology over a period of 12 months, evaluating incision types, chest closure technique, and number of drains with drainage duration. The patients were divided into three groups: peri-costal (PC), proposed extra-costal (EC), and simple (SC) suture, respectively. Pain was measured on day 1, 2, 5, 7, and at 6 months post-operatively using the Visual Analogic Scale. Results: No significant differences in pain level were recorded in the first two post-operative days between the PC and EC groups. However, a significant decrease in pain level was observed on day 5 and at 6 months post-operatively, with a mean level of 3.5 ± 1.8, 1.2 ± 1 for the EC group compared to a mean value of 5.3 ± 1.6, 3.2 ± 1.5, respectively. No significant differences were observed regarding other evaluated variables. Conclusions: The lower recorded pain scores in patients with extra-costal chest closure are a strong argument to use this technique. Its ease of use is similar to the classic peri-costal closure, and the time needed to perform it is not significantly increased. The association of this technique with less invasive procedures and short drainage duration limits chronic post-operative pain. This procedure may represent an option for decreasing healthcare costs associated with the management of PTPS.

## 1. Introduction

A surgeon has to deal over the course of his career not only with the specific pathology of his patients, but also with the management of pain. Thoracic incisions are known to be amongst the most painful surgical approaches, generating acute post-operative pain, but also chronic pain, with frequencies varying between 40% and 60% [1,2,3]. This leads to a poor quality of life for thoracic surgery patients, and in some cases many patients require a further surgical procedure—intercostal neurectomy to attenuate the pain. For these patients, prolonged antalgic medication is required. Almost always thoracic approaches involve thick nerves—the intercostal nerves, that are wounded directly or indirectly at incision site, by the use of rib retractors or rib fractures at thoracotomy. Usually, nerve involvement includes more than one nerve, at least 2 intercostal nerves can be affected: the nerve corresponding to the thoracotomized intercostal space and one or two nerves at the chest drainage site. Furthermore, depending on the modality of chest closure the nerves adjacent to the thoracotomy may be involved.

The most widespread thoracotomy closure technique is the placement of one or two peri-costal stitches, because it is a fast and easy technique [4]. In most situations these stitches are tightened excessively, which leads to pain in the post-operative period. For many patients complaining of chronic pain, a CT scan identifies a very narrow intercostal space corresponding to the thoracotomy incision, and in many cases even bony bridges between ribs, entrapping and compressing the intercostal nerve. The literature describes many methods in treating chronic pain with various results, from epidural catheters, cryoanalgesia, intercostal nerve blockade, pulsed radiofrequency of the dorsal root ganglion chronic use of non-steroidal anti-inflammatory drugs (NSAIDs) or opioid medication [5,6,7,8]. Chronic pain, thus, has a polymorph origin, and its management implies additional costs for the healthcare system.

This paper describes an alternative thoracotomy closure technique intended to minimize chronic post-operative pain by minimizing pressure on the intercostal nerve, suited to close the chest in selected cases.

## 2. Materials and Methods

For the purpose of this study, we included all patients that underwent a thoracic surgery procedure that required a thoracotomy, mini-thoracotomy, or video-assisted thoracic surgery (VATS) approach, admitted to our ward over a 12-month period. Patients who presented pre-operative pain at the procedure site, previous use of analgetic treatment up to one month pre-operatively, ipsilateral previous thoracotomy, ipsilateral shoulder pain, past history of rib fractures at thoracotomy site, psychiatric illness, or refusal to participate in the study were excluded from our study. All patients underwent various surgical procedures using the following approaches: axillar or posterolateral thoracotomy, axillar mini-thoracotomy, or VATS procedures. Major surgical procedures were performed through an open approach, whereas the VATS approach was indicated for diagnostic procedures, such as pleural lung or mediastinal biopsies and small wedge resections. In all cases the latissimus dorsi muscle was transected, with muscle sparring of the anterior serratus muscle. The intercostal muscles were carefully divided using the electrocautery just at 4 mm from the superior margin of the inferior rib, carefully, not to damage the intercostal vasculo-nervous bundle. Rib spreaders were used for patients with mini-thoracotomy or open thoracotomy, with gradual retraction to spear the rib. After completion of the procedure, one or two soft 24F chest tubes were placed, depending on estimated post-operative air leaks. The anesthesia procedure comprised of general anesthesia with selective oro-tracheal intubation. No local anesthesia was used. The post-operative pain for the patients included in this study was assessed and treated exclusively by the anesthesiology team. Pain was measured using VAS scale (Table 1) recorded on post-operative day 1, 2, 5, 7, and at 6 months.

No peri-operative epidural analgesia was used for any patients. The post-operative pain medication was standardized to all patients. Post-operative pain treatment consisted of 100 mg i.v. Ketoprofenum administered at 8 h, along with 10 mg/mL i.v. Acetaminophen if the patients had good liver function in the first 5 post-operative days. If the liver function was abnormal, due to associated pathology, Nefopamum 20 mg/mL was administered slowly i.v. three times/day, and if the recorded pain scores remained high the dose of nefopanum was increased up to a maximum value of 120 mg/day.

**Figure 1:**
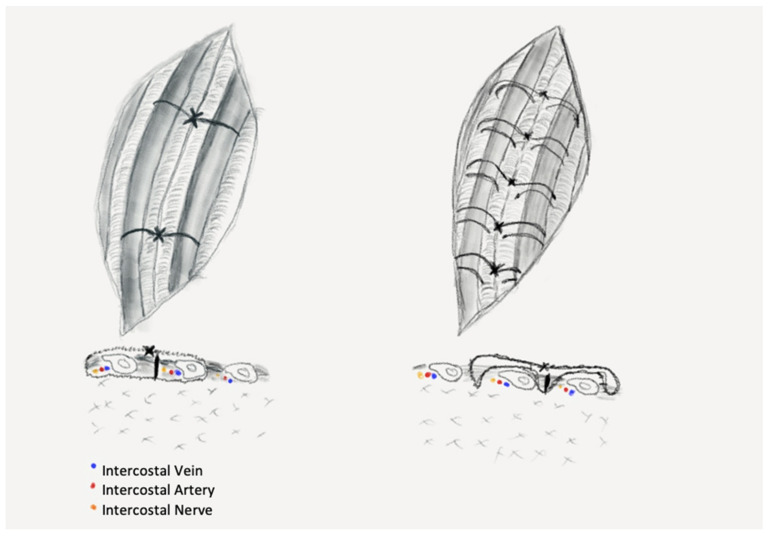
Final aspect of the closed thoracotomy: left—standard peri-costal closure (two intercostal bundles are entrapped in the suture line); right—extra-costal closure (the suture line spares both the vessels and nerves).

We divided the patients into three groups: PC—peri-costal chest closure; EC—extra-costal chest closure; and SC—simple closure. Randomization was performed for patients undergoing open procedures with regard to type of closure (PC vs. EC), patients surgically treated through VATS approach underwent simple closure, being considered as the control group and, therefore, no randomization was performed in this group. Half the patients were operated on in 2019, then the study was interrupted because of the outbreak of SARS-CoV2 pandemic and was resumed in April 2020 when the special protocols and in-hospital circuits were established, and a normal surgical activity was resumed. The PC group comprised of patients who had their chest closed in a standard fashion by using sutures passed around the ribs, in a peri-costal way (Figure 1—left): two sutures placed evenly along the length of the thoracotomy, through the intercostal muscles at the upper margin of the ribs adjacent to the incision.

The EC group of patients received chest closure through a novel stitching procedure using extra-costal sutures, a procedure that was introduced in our clinic to obtain less pressure on the intercostal space. The suturing technique comprises of the following steps: one peri-costal suture is passed through the middle of the incision and is used to approximate the ribs. This stitch is removed after placement of the extra-costal sutures, as shown in the figure below (Figure 1—right).

**Figure 2:**
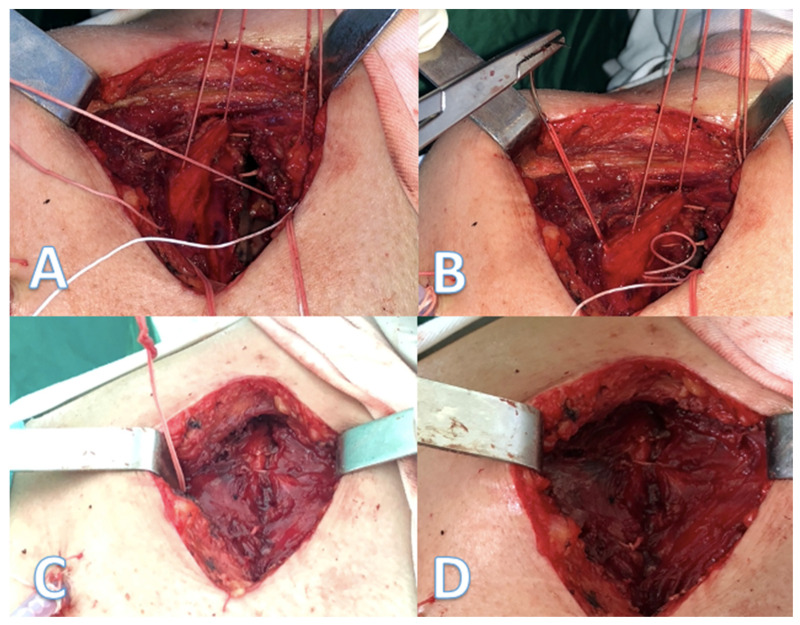
(**A**,**B**)—after the rib approximation peri-costal is in place stitches are passed through the intercostal muscles at 1/1.5 cm each; (**C**)—after approximation of the thoracotomy the extra-costal stitches are knotted together and the peri-costal is removed; (**D**)—final aspect.

We placed the extra-costal stitches from the back to the front of the incision with 1–1.5 cm spacing between stitches (Figure 2A,B), one end was passed in and out through the intercostal muscles of the upper neighbor intercostal space, then through the intercostals of the thoracotomized space and the other end in and out of the lower neighbor intercostal muscles. Then, the approximation of the thoracotomy was performed by closing the peri-costal, followed by closing all the extra-costal stitches and, finally, the peri-costal suture used for approximation of the incision was removed (Figure 2C,D). After removal of the peri-costal suture, the thoracotomy incision relaxes, resulting in a slight distancing of the adjacent ribs, thus ensuring a lesser compression of the intercostal nerve.

The third group, SC (n = 90), benefitted by simple muscular sutures due to small incisions. All these patients underwent VATS procedures; thus, no rib retractor was used.

The institutional review board of our university approved this study (No. 3880/4 February 2019), all patients were informed regarding the purpose of the study and written consent was obtained prior to surgery; refusal to participate lead to exclusion from our study. All 3 groups were blinded to what chest closure technique was used. A minimum of 80 patients/group was targeted to ensure significant differences between recorded pain and to prevent losing track of the patients in the follow-up period.

Statistical analysis was performed using SPSS software for Windows version 21 (IBM Corp., Armonk, NY, USA). Descriptive analysis of continuous data was presented as mean and standard deviation. Pearson’s chi square test, one- and two-way ANOVA tests were used for the assessment of correlation between evaluated groups. A *p* value of <0.05 was considered statistically significant.

## 3. Results

A total of 311 patients were enrolled in this trial and divided into three groups depending on suture closing technique PC (n = 113), EC (n = 108), and SC (n = 90). Demographic data are summarized in Table 2, along with data related to the procedure type, chest drain number, duration of chest drain maintenance, and mean hospital stay. Although statistically significant differences were recorded for the whole study group for the procedure length and mean number of chest drains, post hoc analysis revealed that no significant differences were present between PC and EC groups. Additionally, no significant differences in prolonged air leakage were observed between the aforementioned groups; prolonged air leakage being considered a complication with a possible impact on long term pain, due to maintaining chest drainages for a prolonged period.

There were no significant differences between the patients in group PC and EC, in terms of gender distribution, type of surgical procedure, or length of surgery. Only two deaths were recorded in the immediate post-operative period, both in the PC group, being unrelated to the chest closure type. No post-operative wound infection, bleeding or thoracic herniation was recorded for the study group.

**Figure 3:**
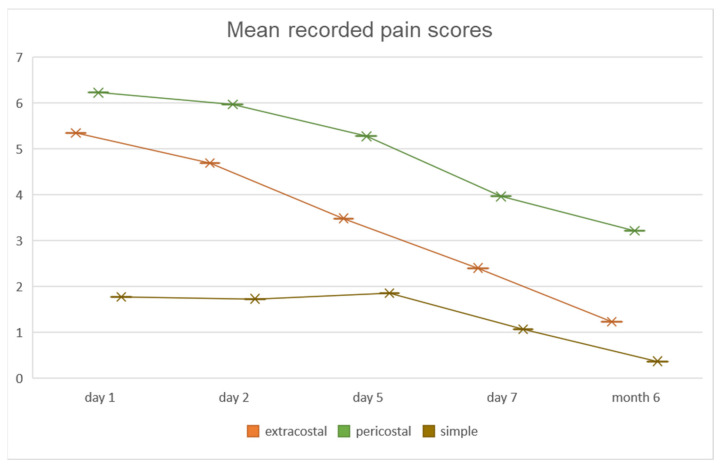
Recorded mean pain scale values, showing lower values in the EC vs. PC groups.

Post-operative pain level was assessed at day 1, 2, 5, 7, and at 6 months, respectively (Figure 3). No statistically significant differences of mean VAS scores were observed between PC and EC patients in the first two post-operative days (day 1—PC: 6.2 ± 1.6 vs. EC: 5.4 ± 1.5; day 2—PC: 5.9 ± 1.6 vs. EC: 4.7 ± 1.7). A significant decrease in pain level was observed in the EC group compared to the PC patients on day 5 and 7, respectively (*p* < 0.001). On the 5th post-operative day, mean pain level for the PC group maintained at 5.3 ± 1.7, whereas for patients in the EC group mean value of VAS decreased to 3.5 ± 1.8. Lower values were recorded for both groups on the 7th post-operative day, however with a higher mean value in the PC group (4 ± 1.6) compared to that of EC patients (2.5 ± 1.7). In the SC group, considered as a control group, surgical approach was most commonly performed through VATS, these patients having significantly smaller incisions (max. 3 cm) compared to the other study groups, and thus recording significantly lower pain scores at all time points (*p* < 0.001). Regarding post-operative pain management, aside from standard pain medication, opioid treatment was reserved to patients (n = 45) with VAS scores higher than 8, until the recorded pain value went below 6. If the use of opioids did not have the estimated effect in pain relief, we applied local anesthesia to the thoracotomized intercostal space, and also to both spaces above and below the thoracotomy (n = 3).

**Figure 4:**
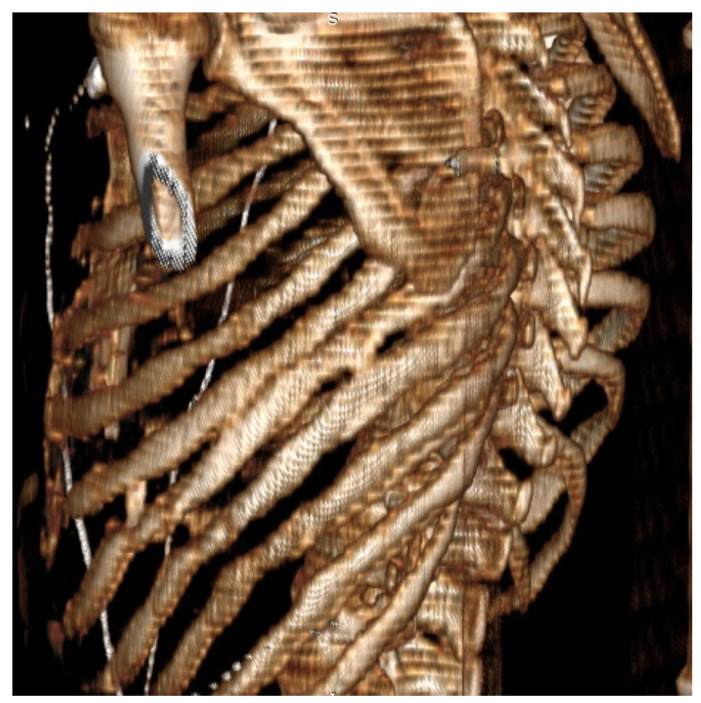
Computed tomography scan 3D reconstruction at 6 months after surgery in a patient from group PC with recorded pain value of 8 showing narrowing of the thoracotomized intercostal space and bony bridges between neighbor ribs entrapping the intercostal nerves.

Pain levels at 6 months following surgery recorded significantly higher values in PC patients (3.2 ± 1.5) than those recorded in the EC group (1.2 ± 1, *p* < 0.001). Patients with persistent high scores (>7 on VAS) at 6 months after surgery were evaluated through imaging studies to evaluate potential causes for the pain. Patients in the PC group presented on computed tomography scans with narrowing of the intercostal spaces and bony bridges, as shown in Figure 4, most likely being the underlying cause for the persistence of intercostal pain. No patients in the EC group presented with a VAS score higher than 4 at 6 months following surgery.

## 4. Discussion

Chronic post-operative pain still remains an important issue for the patients, even in the VATS era. It affects their quality of life and prevents them from carrying on with their daily activities. Once present, chronic pain presents a significant challenge, recovery and pain management are complicated by multiple factors. The presence of important comorbidities further limits therapeutic options. Chronic pain should be treated with multimodal regimens, tailored to the patient and procedure [9,10]. This leads to further admissions to the hospital and further related costs.

Furthermore, although the majority of countries are now in the VATS era, and VATS techniques are proven to be less painful, in many low-income or mid-income countries these techniques are limited to diagnostic procedures. Surgical procedures, such as lung resections in these countries, are performed mainly by an open approach. This aspect has represented the motivation to perform this study.

The causes for PTPS are not fully understood by the scientific community. The literature indicates, as a possible cause for chronic pain, the intercostal nerve damage, apparition of degeneration, axonal sprouting or neuromata, occurring both intraoperative and post-operative, generating hyperalgesia and allodynia [11], describing it as “neuropathic” [12]. It has been shown that the neuropathic component is responsible for only half of the patients [13]. Pain uses both nociceptive and neuropatic mechanisms with somatic afferences from the intercostal nerves, by division of the chest wall, pleura, lung parenchyma, and hilar structures, and visceral afferences from manipulation of the vagus and phernic nerves or by division of the visceral pleura and pericardium. Impulses are transmitted centrally to the limbic system and somato-sensory cortex, followed by the release of inflammatory mediators responsible for the primary sensitization. Further signals lead to a hyperexcitability of the dorsal horn neurones and higher pain centers through activation of N-methyl-D-aspartate (NMDA) receptors which causes central sensitization and chronic pain [14].

However, the main event causing chronic pain is intercostal nerve damage, partly related to rib fractures at thoracotomy, partly to incision types (open vs. VATS) and lengths, usage of rib retractors. It is known that rib fractures are associated with higher pain scores on the long term [15,16]. That is why we recommend rib protection while using a retractor with soft material, placed in-between the rib and the retractor. The surgical technique is ultimately of paramount importance. Although it has been shown that using the VATS technique minimizes the risk of PTPS compared to open surgery regarding peri-operative pain [17], there are studies showing that in terms of chronic pain there are no significant differences between standard and less invasive techniques at one year after surgery [18].

However, not only the approach and procedure type planning are important, the chest closure has a key role in pain prevention [19]. If the ribs are approximated too tight this leads to direct nerve pressure. There are many ways to close the chest described in the literature. Cerfolio et al. described a method of closure by drilling holes in the lower rib and approximating the intercostal by sutures passed through the holes [12], and although this method is reported as successful in reducing post-operative pain [20,21], it is both expensive and has a risk in harming the underlying lung parenchyma. Additionally, it takes an increased amount of time to prepare the drill setup. El-Hag-Aly et al. published a paper describing the “double-edge closure”, which was, in fact, a modified “edge closure” described by Sakakura [22]. The double-edge technique passes the stitches underneath the lower division of the thoracotomized intercostal, keeping the upper division of the muscle inside the thorax to protect the intercostal nerve. It eliminates the disadvantages of using drills, having shorter times for setup and execution and the risk of lung damage with the drills. The difference between the peri-costal suture and El-Hag-Aly’s method is that pain is reduced by passing the sutures between the rib and the intercostal bundle. To achieve these multiple dissections of at least two intercostal spaces are required, increasing the risk for iatrogenic bundle damage.

Our extra-costal closure is very easy to execute, requiring only a few minutes, and no further dissection being necessary, since the sutures are passed only in and out through the intercostal muscles, at distance from the intercostal nerves. Uniform placement of these stitches ensures an even distribution of pressure on the thoracotomized space and prevents lung herniation, making it possible to be used for even long thoracotomies, while the removal of the approximation wire relaxes it, keeping low pressure on the nerve. In our cohort we had no lung herniation. One downside of our closure is that careful division of the intercostals associated with soft material protection in the event rib spreader usage, in order to preserve intercostal muscle mass, are the key to ensure the technique’s success and efficiency in terms of pain. Any damage to the intercostal muscles could lead to impossibility of applying the extra-costal closure.

We showed that extra-costal suture, if performed properly, minimizes pain scores at 6 months post-op., with a mean of 1.8 points versus the standard peri-costal technique.

The patients in the SC group generated lower pain scores because the incisions used were smaller (max. 3 cm) and no rib retractors were placed. Additionally, for these patients the chest drain was placed in the same intercostal space used for the surgical procedure, thus eliminating compression of different nerves. Pain at 6 months, although of lower intensity was still present even is this group.

Because pain is, by nature, subjective, and each individual has his own perception of pain intensity, it is a very difficult symptom to quantify. For this reason our study has been performed in only one department, using the same surgical teams, but, far more importantly, the same anesthesiology team, who managed all the patients in the first post-operative days. The surgical techniques used were standardized, with extra focus on the chest closure technique. Pain medication was uniform in all patients, to create similar post-operative conditions. The group selection was well matched and all the patients were blinded to the chest closure technique used.

In spite of all this, we identified some very important limitations in our trial. One limitation is that in our attempt to make the pain objective we used pain scales, which provide subjective data recorded from the patients. We observed that many patients had difficulties in providing an exact number corresponding to pain from the pain scale and many patients with lower scores asked for pain medication, while others with higher scores were reluctant to use painkillers. Additionally, due to the fact that in our country the health system is under-financed, correlated with high cost of the endo-staplers and endoscopic consumables, the VATS techniques are very limited to diagnostic procedures, such as pleural biopsy, mediastinal lymph node biopsy, and the procedures with curative purpose were limited to wedge resections for benign disease—tuberculomas, emphysematous disease. Thus, including more ample surgical procedures in the VATS group was not possible and may determine to a certain degree bias in recorded pain values.

Another possible limitation is the fact that the study had a gap caused by the SARS-CoV2 pandemic, which could have had an impact on pain data recorded from our patients, who might have reported lower pain scores to avoid direct prolonged contact with the personnel and to minimize in-hospital stay for fear of the virus.

As we have shown, extra-costal suture of the chest is a reliable technique compared to the classic closure type. Although it is not appliable in all cases. The patients that are candidates for this technique must be selected carefully, and the surgery and post-operative plan must be established very clearly, in a personalized fashion.

Our study shows that extra-costal closure has good results in terms of chronic pain, but the cohort had strict inclusion criteria. The day-to-day hospital activity brings in front of the thoracic surgeon many people, fundamentally different as individuals, exceeding the inclusion criteria of this study. Some patients have a history of anxiety, which has been proven to be associated with higher recorded pain scale values, so they would benefit well from this less painful closure type. Additionally, the patients’ anatomy is particular for each case, some of the patients have thick intercostal muscles, but many patients have debilitating diseases that lead to poor nutrition and therefore poor muscle quality. The extra-costal suture is a muscular suture as described in this paper, so poor muscle quality has a very high risk of developing intercostal muscle rupture and consequent lung herniation, although in our cohorts we recorded none such event. If rib fractures occur at thoracotomy after applying the retractor the recorded pain values are higher, because the pain is bone related rather than produced by nerve compression due to peri-costal sutures, so these patients do not benefit by the advantages provided by the extra-costal suture.

So, the choice of the extra-costal closure must be tailored to each individual patient, taking into account educational and psychological factors, patient anatomy, surgery related incidents (rib fractures, reinterventions) that could make the surgeon change the surgical strategy “on-the-go”.

## 5. Conclusions

In conclusion, the extra-costal chest closure is a cheap, easy and fast, safe and effective in reducing the apparition of chronic PTPS. This technique was initially designed for patients with small thoracotomies, but we have proven that it is safe to use even for longer incisions, if the patients anatomy allows it (thick intercostal muscles). The procedure’s success is proved by our study’s results and the multiple advantages with virtually no long-term complications, along with the possibility to apply it even in the case of long thoracotomies fully justifies its use. The choice of using this technique over the classic closure should be made for each patient in a personalized fashion, if there are no rib fractures when performing the thoracotomy.

## Figures and Tables

**Table 1:** Visual analogic pain scale used for the purpose of this study.

0	1	2	3	4	5	6	7	8	9	10
No pain	Mild pain			Moderate Pain			Severe Pain			Worst Pain Possible

**Table 2:** Demographic and procedure related data of the patients.

	Pericostal Group (PC) n = 113	Extracostal Group (EC) n = 108	Simple Closure Group (SC) n = 90	*p* Value
Age (years)				
Mean ± SD	56.1 ± 13.0	54.7 ± 14.0	57.4 ± 14.9	*n.s.*
Gender				
Male	70	51	67	*n.s.*
Female	43	38	41	
Types of operation				
Biopsy	6	6	76	*n.s.*
Wedge	45	41	4	
Lobectomy	13	13	0	
Pneumonectomy	7	6	2	
Decortication	15	13	3	
Excizion/Enucleation	9	6	0	
Rib resection	0	0	2	
Re-thoracotomy for bleeding	0	2	0	
Other	19	20	5	
Procedure length (hrs. dec.)	2.7 ± 1.7	2.6 ± 1.4	1.0 ± 0.4	<0.001
Drainage				
Mean number of chest drains	1.5 ± 0.6	1.6 ± 0.5	0.9 ± 0.2	<0.001
Mean duration of chest drains (days)	3.9 ± 6.7	4.2 ± 4.7	2.8 ± 2.7	*n.s.*
Length of stay (days)	13.4 ± 10.6	10.1 ± 6.9	7.1 ± 3.9	0.001
Deaths	2	0	1	*n.s.*

*n.s*. = not significant, *p* > 0.05.

## Data Availability

The data presented in this study are available on request from the corresponding author. The data are not publicly available due to the fact that the database contains patient personal data.
